# The Impact of Matrix Metalloproteinase 2 on Prognosis and Clinicopathology of Breast Cancer Patients: A Systematic Meta-Analysis

**DOI:** 10.1371/journal.pone.0121404

**Published:** 2015-03-27

**Authors:** Yiping Chen, Xiaochen Wang, Guodi Chen, Caixia Dong, Depu Zhang

**Affiliations:** 1 Department of Psychiatry, The Second Affiliated Hospital, Zhejiang University School of Medicine, Hangzhou, PR China; 2 Department of Surgical Oncology, The Second Affiliated Hospital, Zhejiang University School of Medicine, Hangzhou, PR China; 3 Department of Radiation Oncology, The Second Affiliated Hospital, Zhejiang University School of Medicine, Hangzhou, PR China; 4 Department of Medical Oncology, The Second Affiliated Hospital, Zhejiang University School of Medicine, Hangzhou, PR China; 5 School of Medicine, Shandong University, Jinan, Shandong, PR China; Centro di Riferimento Oncologico, IRCCS National Cancer Institute, ITALY

## Abstract

**Backgrounds:**

Matrix metalloproteinase 2 (MMP-2) plays a crucial role in the progression of breast cancer (BC). The prognostic role of MMP-2 expression in BC patients has been widely reported, but the results were inconsistent. Thus, a meta-analysis was conducted to gain a better insight into the impact of MMP-2 expression on survival and clinicopathological features of BC patients.

**Methods:**

Identical search strategies were used to search relevant literatures in electronic databases update to August 1, 2014. Individual hazard ratios (HRs) and odds ratios (ORs) with their 95% confidence intervals (CIs) were extracted and pooled to evaluate the strength of the association between positive MMP-2 expression and survival results and clinicopathological features of BC patients. Begg’s tests, Egger’s tests and funnel plots were used to evaluate publication bias. Heterogeneity and sensitivity analysis were also assessed. All the work was completed using STATA.

**Results:**

Pooled HRs and 95% CIs suggested that MMP-2 expression had an unfavorable impact on both OS (HR: 1.53, 95% CI: 1.29–1.82) and DFS/RFS/DDFS (HR: 1.41, 95% CI: 1.07–1.86) in BC patients. Furthermore, MMP-2 expression was significantly associated with lymph node metastasis (positive vs negative: OR 1.91, 95% CI 1.17–3.12).

**Conclusion:**

In conclusion, positive MMP-2 expression might be a significant predictive factor for poor prognosis in patients with BC.

## Introduction

Breast cancer (BC), one of malignant tumors that jeopardize women’s health seriously, is the leading cause of mortality in females worldwide. It affects 1.38 million individuals worldwide every year [1]. In China, the incidence of breast cancer ranks first in female cancers and the mortality shows an upward trend during the past 30 years[2]. Although great progress has been made in the treatment strategies for BC, which significantly changed patient outcomes, BC is still one of the most frequent malignancies with a poor prognosis.

Prognostic factors for recurrence and progression of BC could help clinicians identify new diagnostic and therapeutic techniques to improve the patients’ quality of individual care [3]. Traditional prognostic factors for patient survival include tumor size, lymph node status, distant metastasis, TNM stage, histological grade, and hormone receptor status [4–6].

Unfortunately, most potential prognostic biomarkers are relatively crude measures and it is insufficient to predict the choice of optimal treatment for an individual. Thus, it is indispensable to identify precise biomarkers with predictive value for the survival of patients undergoing treatment.

BC mortality derives overwhelmingly from invasion and metastasis. Matrix metalloproteinases (MMPs) are a family of enzymes which play important roles in tumor invasion and metastasis [7]. Extracellular matrix (ECM) and basement membrane together form the first vital barrier in the course of tumor metastasis and type IV collagen is the main component of it. Matrix metalloproteinase 2 (MMP-2), a main member of MMPs, is thought to be the key enzyme for metastasis of tumor with the physiological action of degrading type IV collagen [8–11].

High levels of MMP-2 expression are often correlated with clinicopathological features of BC, such as tumor size, lymph node metastasis, distant metastasis, histological grade and hormone receptor status, but the conclusions remain controversial[12–22]. Similarly, a number of studies investigated the impact of MMP2 expression on the survival of patients with BC, but no consistent results were reported. Some authors reported that MMP-2 expression was associated with poor prognosis [14, 17, 19, 23], but others failed to give the same results [15, 18, 24, 25]. The discrepancies in conclusions may be attributed to some of the defects in individual study such as small sample size or low statistical power. Thus, the combination of these data to systematically evaluate the prognostic value of MMP-2 expression in BC patients is warranted at present.

The current study aims to gain a better insight into the impact of MMP-2 expression on survival and clinicopathological features of breast cancer patients using a meta-analysis. The results showed that expression of MMP-2 was associated with poor survival and lymph node metastasis, suggesting that postoperative detection of MMP-2 expression in breast cancer would help develop better therapy strategies, distinguish high risk populations from the patients undergoing surgery and make better follow-up plans.

## Methods

### 2.1 Search strategy and study selection

Relevant articles assessing associations of MMP-2 expression and survival outcomes or clinicopathological features in patients with BC were extracted from PubMed, Ovid, EMBASE, Web of Science and China National Knowledge Infrastructure (CNKI) database update to August 1, 2014. The search strategy used the following combined terms: (“breast cancer” or “breast neoplasm” or “breast carcinoma”) and (“MMP-2” or “MMP2” or “matrix metalloproteinase-2”, or “type IV collagenase” or “gelatinase-A”). No lower date or language limits were applied initially, but for full-text review and data analysis, only papers in English or Chinese were included finally. In order to minimize the deviation caused during the search process, references in all relevant articles were scanned to identify other potentially applicable reports.

This meta-analysis gathered complete and comprehensive data from published studies dealing with the prognostic implication of MMP-2 protein expression (not mRNA) in tumor tissue (not serum or plasma) by immunohistochemistry (IHC) in patients with primary BC who underwent surgical resection. Studies on associations of MMP-2 expression and survival outcomes or clinicopathological features were screened respectively. For MMP-2 expression and survival outcomes, the additional criteria must be met in order to ensure the high quality of this article: (1) it was a full paper that assessed the association between MMP-2 expression and clinical outcomes (overall survival (OS), disease free survival (DFS), recurrent free survival (RFS), distant disease free survival (DDFS)) in breast cancer; (2) hazard ratio(HR) and 95% confidence interval (95% CI) could be obtained from the articles or calculated based on the information in the paper with methods developed by Parmar [26], Williamson [27], and Tierney [28]. For MMP-2 expression and clinicopathological features, the additional criteria were: (1) the association between MMP-2 expression and clinicopathological features in BC was assessed; (2) data in the article was available for calculating odds ratio (OR) and 95% CI.

Study was excluded based on any of the following criteria: (1) it was review, letter or experiment on animal models; (2) it lacked key information for HR or OR estimation analysis; (3) it mentioned the MMP-2 polymorphism instead of expression. When an individual author reported two or more publications on possibly the same patient populations, only the most recent or complete study was included.

The confirmation of qualified literature was divided into two steps. First, we screened the title and abstract to see which articles needed further review. Second, the entire article was reviewed to ascertain whether it can be included in this meta-analysis. Two reviewers (Yiping Chen and Xiaochen Wang) independently determined study eligibility. Disagreements were resolved by consensus.

### 2.2 Survival outcomes and clinicopathological features used in the meta-analysis

OS and data relevant to the relative recurrence risk of breast cancer were recognized as the clinical outcomes for our meta-analysis of the association between MMP-2 expression and prognosis in BC patients. Many studies have assessed the OS and DFS outcomes or alternatively the RFS/DDFS. The definition of each outcome was as follows: OS, time from the date of diagnosis to death or the end of the follow up; DFS, time from the date of diagnosis to the first distant metastasis or local relapse from BC or the end of the follow up; RFS, time from the date of diagnosis to a recurrent BC or the end of the follow up; DDFS, time from the date of diagnosis to the first distant metastasis or the end of the follow up. The clinicopathological features referred to the TNM stage, tumor size, lymph nodes status, distant metastasis, histological grade, estrogen receptor status and progesterone receptor status. The TNM stage, tumor size, lymph nodes status and distant metastasis were determined according to the TNM staging classification for BC. The histological grades were assessed according to Scarff-Bloom-Richardson histological grading system [29, 30].

### 2.3 Data extraction

Data extracted from the articles included: name of first author, publication year, country, median age of patients, median follow-up time, study sample size, the percentage of MMP-2 positive, definition of MMP-2 positive (cut-off), location of MMP-2 expression in tissue, survival outcomes, method of HR estimation, method of survival analysis, HR and 95% CI, clinicopathological features involved, OR and 95% CI. If the above information were not mentioned in the original study, the item was treated as “not reported (NR)”. Inconsistencies in the research process were resolved through debate and consultations.

### 2.4 Quality assessment

Study quality was assessed in duplicate independently by two investigators (Yiping Chen and Guodi Chen) according to the Newcastle-Ottawa quality assessment scale (NOS) as recommended by the Cochrane Non-Randomized Studies Methods Working Group[31–33]. This scale was developed to assess the quality of nonrandomized studies, specifically cohort and case-control studies. According to the NOS, studies were assessed based on three broad perspectives: patient population and selection, study comparability and outcome of interest. We considered studies that got six or more scores of the NOS criteria as high quality [33, 34].

### 2.5 Statistical analysis

HRs and 95% CIs were combined as the effective value to measure the impact of MMP-2 expression on survival of BC patients. Some of the included studies provided HR and 95% CI directly. When these statistical variables were not given explicitly, we calculated them from available data using the methods reported by Parmar [26], Williamson [27], and Tierney [28]. The available data referred to the total number of events, the number of patients in each group, the log-rank statistic and its P-value or the O-E statistic (difference between numbers of observed and expected events). If the only existing survival data were in the form of figures, the Kaplan-Meier survival cure was read by Engauge Digitizer version 4.1 (free software down-loaded from http://sourceforge.net) by two independent investigators (Yiping Chen and Caixia Dong) to reconstruct the HR. Additional information and data needed for meta-analytic calculations were obtained by emailing the authors when even the available data or Kaplan-Meier survival cure could not be obtained from the articles. Furthermore, when univariate and multivariate analysis were both obtainable, the latter was chosen to be pooled because survival result in BC was affected by multiple factors.

The association between MMP2 expression and clinicopathological features was assessed using combined OR with its 95% CI. The key exposure variable was positive or negative of MMP-2 expression, and the preferred control group was negative. The outcome variable of interest was defined as the presence or absence of high TNM stage, larger tumor size, lymph nodes invasion, distant metastasis, higher histological grade, estrogen receptor positive expression and progesterone receptor positive expression. Combined HR or OR > 1 with their corresponding 95% CIs not overlappling 1 implied a worse survival for the group with positive expression of MMP-2 or the significant association between expression of MMP-2 and clinicopathological characteristics.

Heterogeneity across studies was evaluated by Chi-square-based Q statistical test. P>0.10 for the Q-test indicated a lack of heterogeneity among the studies. Meanwhile, the I^2^ statistic (I^2^ = 0–40%, no or moderate heterogeneity; I^2^ > 40%, significant heterogeneity) was also calculated to quantify the proportion of the total variation due to study heterogeneity [35]. Fixed-effect model was used if there was no significant heterogeneity. Otherwise, the random-effect model was used. Potential publication bias was assessed by visual inspection of the funnel plots and was further assessed by Begg’s funnel plots and Egger’s linear regression test [36] in which P-value <0.05 was considered to indicate statistical significance. Sensitivity analysis was performed by sequential omission of individual studies to examine the stability of the pooled results.

Statistical calculations were all performed using STATA version 12.0.

## Results

### 3.1 Study characteristics

**Fig 1:**
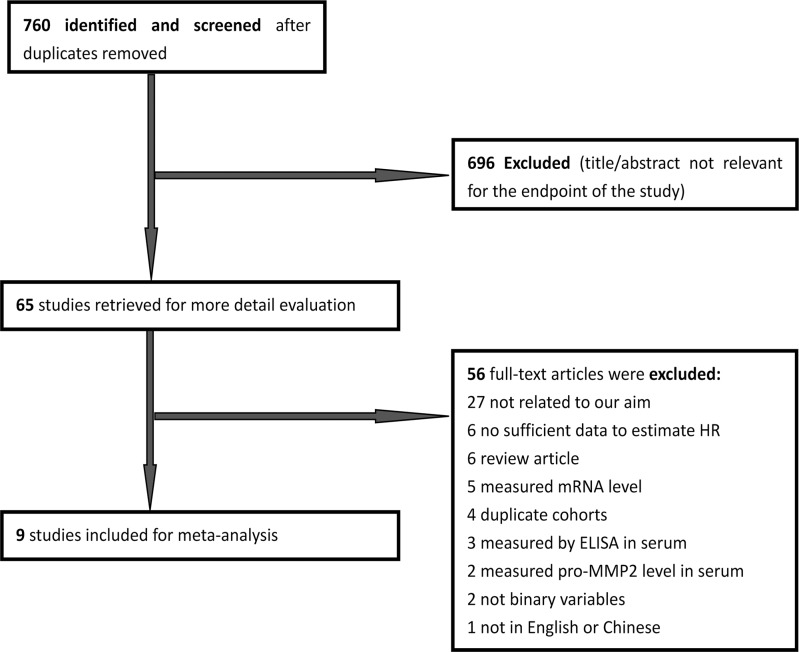
Literature search and selection of articles for survival outcomes.

**Fig 2:**
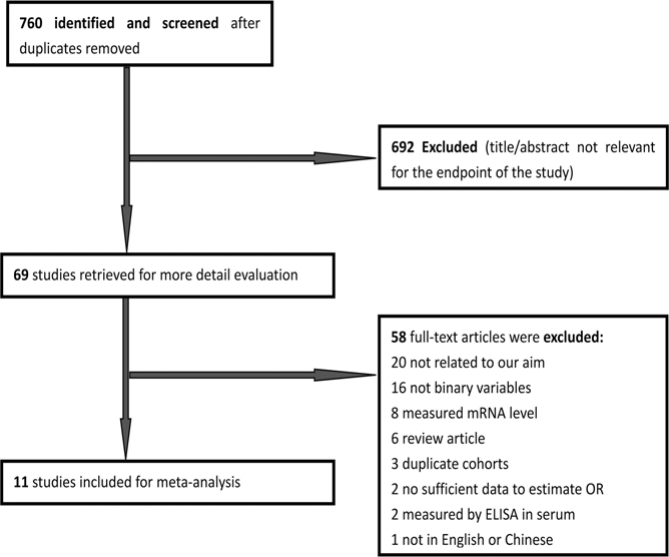
Literature search and selection of articles for clinicopathological features.

A total of 760 papers potentially eligible for inclusion were confirmed with the initial search strategy mentioned above. After excluding irrelevant studies, overlapping studies and those without information to calculate the HR or OR, 9 articles finally met the inclusion criteria for MMP-2 expression and survival outcomes [14, 15, 17–19, 23–25, 37](Fig. 1). For MMP-2 expression and clinicopathological features, 13 articles were included [12–22] (Fig. 2). What had to be declared was that as two articles [16, 20] provided information about MMP-2 expression in tumor cells and stroma cells, we processed each one independently as two studies in the meta-analysis.

The major characteristics of retained studies included for survival outcomes were summarized in Table 1. Four studies evaluated patients from China, two from Finland, one from Netherlands, one from Greece and one from Croatia. There were eight studies utilizing OS to assess the prognostic value of MMP-2 expression in breast cancer patients and four studies using DFS/RFS/DDFS as the indicator. A totally of 1614 patients were included and the number of patients in each study ranged from 66 to 453. Four studies defined the cut off value by complex score combining intensity and percentage of MMP-2 expression, while other studies only used the percentage to define positive expression with the cut off value that varied from 1% to 5%. HR and 95% CI were obtained from the original articles directly in five studies, and data were calculated based on the available information in three individual studies. The remaining one paper, HR and 95% CI for OS were provided directly and that for RFS had to be calculated based on the available information. One article got 9 NOS scale, five got 8, two got 7 and one got 6.

**Table 1:** Characteristics of eligible studies for survival outcomes in meta-analysis.

*Author*	*Year*	*country*	*Recruitment time*	*Age Median (range)*	*Median Follow-up (months)*	*N*	*POSI (%)*	*Tissue*	*Cut-off*	*Event*	*HR estimate*	*Survival analysis*	*HR(95%CI)*	*Quality stars (NOS)*
*Djonov*,*V*.	2002	Greece	NR	58(NR)	NR	94	NR	T	CS	OS	HR	M	1.92(0.53–7.14)	6
										DFS	HR	M	1.43(0.59–3.57)	
*Fan*,*S*.*Q*.	2003	China	May. 1990-Jan. 2001	54(26–68)	30.5(6–120)	66	71.20	T	CS	OS	A	U	2.57(1.08–6.10)	8
*Talvensaari-Mattila*,*A*.	2003	Finland	1981–1995	52(26–85)	NR(60–150)	453	78.10	T	≥1%	OS	HR	M	1.78(1.10–2.88)	8
										DFS	A	U	1.42(1.00–2.02)	
*Hirvonen*,*R*.	2003	Finland	1988–1991	NR(26–85)	NR(117-NR)	137	83.20	T	≥1%	OS	A	U	3.50(0.90–13.54)	7
*Li*,*H*.*C*.	2004	China	1990–1998	NR	61(21–120)	270	56.70	T	≥1%	OS	HR	M	2.86(0.49–16.72)	8
										RFS	HR	M	1.73(0.55–5.4)	
*Zhou*,*S*.	2005	China	Jan. 1996-Dec. 1998	58(41–69)	48(NR)	112	53.60	T	≥5%	OS	HR	M	1.77(1.1–2.85)	8
*Zhang*,*B*.	2008	China	Jan. 1993-Dec. 1993	50.3	92(36–173)	222	23.00	T	CS	OS	HR	U	1.28(0.97–1.68)	7
*Del Casar*,*J*.*M*.	2009	Netherlands	1990–2003	NR(35–60)	65.5(NR)	122	24.60	S	CS	DDFS	HR	U	1.30(0.70–2.20)	8
*Ranogajec*,*I*.	2012	Croatia	Sep. 2002-Sep. 2003	56(29–85)	NR(4–94)	138	55.10	T	CS	OS	A	U	1.46(1.05–2.04)	9

*NR* not reported, *POSI* positive, *T* tumor cells, *S* stromal cells, *CS* complex score combining intensity and percentage, *HR* HR reported in text, *A* HR available data or Kaplan—Meier curves, *U* univariate model, *M* multivariate model.

Table 2 showed the major characteristics of retained studies included for clinicopathological features. Five studies evaluated patients from China, three from Finland, two from Netherlands, one from Germany and one from United States. A totally of 1824 patients were included and the number of patients in each study ranged from 66 to 453. Most of the studies defined the positive expression by the percentage of MMP-2 staining that varied from 1% to 30% except one [21]. MMP-2 positive expression rate was observed ranging from 9.6% to 92.9%. Ten articles contained information of the impact of MMP-2 expression on tumor sizes, nine on lymph nodes status, two on distant metastasis, eight on TNM stage, nine on histological grade, ten on estrogen receptor status and nine on progesterone receptor status. Three studies got 9 NOS scale, five got 8, four got 7 and one got 6.

**Table 2:** Characteristics of eligible studies for clinicopathological features in meta-analysis.

*Author*	*Year*	*country*	*Age/ Median (range)*	*N*	*Positive (%)*	*Location*	*Cut-off*	*T*	*N*	*M*	*S*	*G*	*ER*	*PR*	*NOS*
*Talvensaari-Mattila*,*A*.	1998	Finland	55(NR)	169	84.00	T	≥1%				**√**				8
*Jones*,*J*.*L*.	1999	England	NR	104	36.60	T/S	≥1%		**√**				**√**	**√**	7
*Fan*,*S*.*Q*.	2003	China	53.5(26–68)	66	71.20	T	CS	**√**	**√**		**√**		**√**	**√**	8
*Nakopoulou*,*L(1)*.	2003	Netherlands	57.31(25–87)	135	75.60	T	≥10%	**√**	**√**		**√**	**√**	**√**		7
*Nakopoulou*,*L(2)*.	2003	Netherlands	57.31(25–87)	135	27.40	S	≥10%	**√**	**√**		**√**	**√**			7
*Talvensaari-Mattila*,*A*.	2003	Finland	52(26–85)	453	78.10	T	≥1%	**√**	**√**	**√**		**√**			8
*Hirvonen*,*R*.	2003	Finland	NR(26–85)	137	83.20	T	≥1%	**√**			**√**		**√**	**√**	6
*Li*,*H*.*C*.	2004	China	NR	270	56.70	T	≥1%	**√**				**√**	**√**	**√**	8
*Zhou*,*S*.	2005	China	58(41–69)	112	53.60	T/S	≥5%	**√**	**√**		**√**	**√**	**√**	**√**	7
*Zhang*,*Y*.*G(1)*.	2007	China	54.7(30–78)	92	19.60	T	≥30%	**√**	**√**		**√**	**√**	**√**	**√**	9
*Zhang*,*Y*.*G(2)*.	2007	China	54.7(30–78)	92	55.40	S	≥30%	**√**	**√**		**√**	**√**	**√**	**√**	9
*Sullu*,*Y*.	2011	Germany	52(22–85)	140	92.90	T	CS	**√**	**√**	**√**		**√**	**√**	**√**	8
*Niemiec*,*J*.*A*.	2012	United States	60(37–88)	146	9.60	S	≥1%					**√**	**√**	**√**	9

*NR* not reported, *T* Tumor cells, *S* stromal cells, *T/S* either tumor cells or stromal cells, *CS* complex score combining intensity and percentage, √ data available for calculating OR and 95%CI.

### 3.2 Impact of MMP-2 Expression on OS and DFS/RFS of BC Patients with Subgroup analysis in the meta-analysis

**Fig 3:**
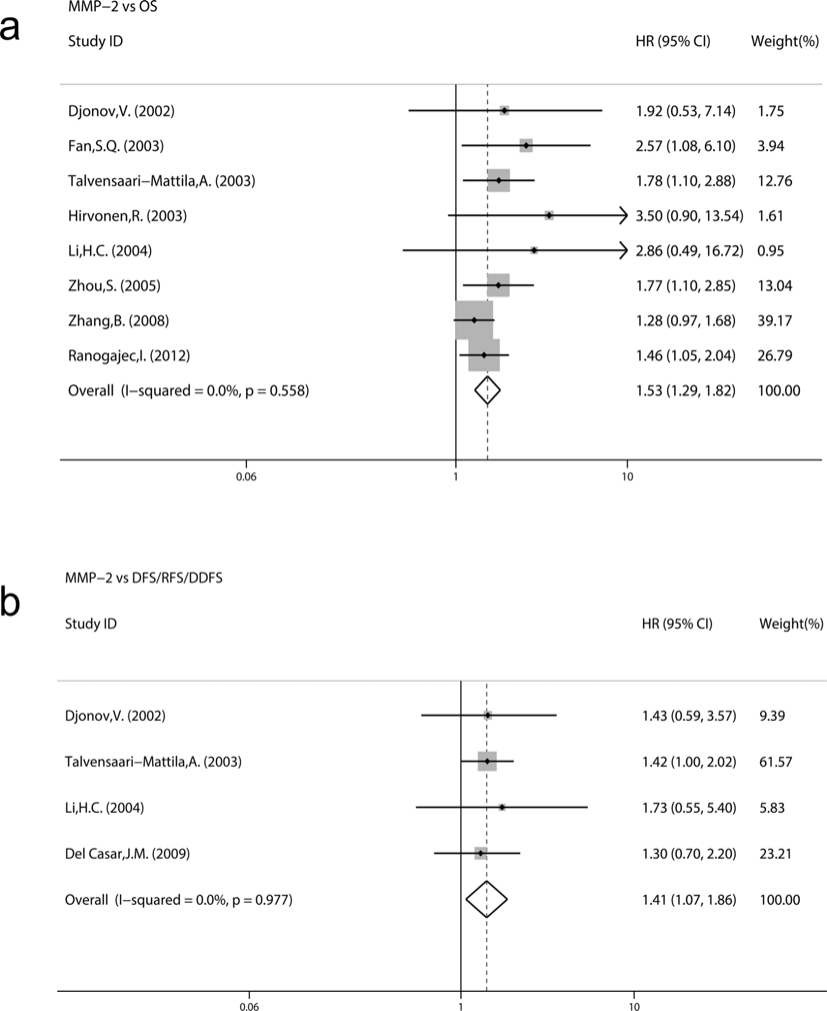
Forest plots of impact of MMP-2 expression on survival. HRs with corresponding 95% CIs of MMP-2 expression on (a) OS and (b) DFS/RFS/DDFS.

Fig. 3 demonstrated forest plots of the HRs and merged results using the fixed-effects model. The meta-analysis was performed on eight studies assessing the association of MMP-2 expression with OS. The pooled HR was 1.53 (95% CI: 1.29–1.82; Z = 4.84; P = 0.000) without heterogeneity (I^2^ = 0.0% P = 0.558) (Fig. 3a). Four studies assessed the association of MMP-2 expression with DFS/RFS/DDFS. The pooled HR was 1.41 (95% CI: 1.07–1.86; Z = 2.43; P = 0.015) (Fig. 3b) without significant heterogeneity (I^2^ = 0.0% P = 0.977). These results suggested that MMP-2 expression was significantly correlated with a worse prognosis of BC patients and that MMP-2 expression was a prognostic factor in BC patients.

Subgroup analysis for OS was performed by the study location, sample size, evaluation standards of positive, methods of HR estimate analysis model and recruitment time. The results indicated that a significant relationship between MMP-2 expression and OS was exhibited in every subgroup (all P > 0.05) without significant heterogeneity (I^2^ ranged from 0.0% to 35.4%, all P >0.05) (Table 3). We didn’t perform a subgroup analysis for DFS/RFS/DDFS because of the small study number involved.

**Table 3:** Subgroup-analysis of the association between MMP-2 expression and overall survival.

***Subgroup***	***No*. *of studies***	***No*. *of patients***	***HR(95%CI)***	***Heterogeneity***		
				χ^2^	P	I^2^ (%)
*Overall effect*	8	1492	1.53(1.29–1.82)	5.85	0.558	0.00
*County*						
*Asia*	4	670	1.47(1.17–1.84)	3.71	0.295	19.00
*Europe*	4	822	1.62(1.25–2.10)	1.83	0.608	0.00
*Sample size*						
*<200*	5	547	1.68(1.31–2.16)	2.83	0.587	0.00
*≥200*	3	945	1.41(1.11–1.78)	1.99	0.369	0.00
*Evaluation standards*						
*percentage*	4	972	1.87(1.36–2.59)	1.14	0.769	0.00
*CS*	4	520	1.41(1.15–1.73)	2.58	0.461	0.00
*HR estimate*						
*Reported in text*	5	1151	1.48(1.20–1.83)	2.87	0.58	0.00
*By estimated*	3	341	1.63(1.21–2.21)	2.71	0.258	26.10
*Analysis model*						
*Univariate*	4	563	1.43(1.17–1.75)	4.08	0.253	26.40
*Multivariate*	4	929	1.81(1.31–2.50)	0.28	0.964	0.00
*Recruitment time*						
*-1995*	3	812	1.43(1.13–1.81)	3.10	0.213	35.40
*1995-*	2	250	1.56(1.18–2.04)	0.42	0.516	0.00

### 3.3 Impact of MMP-2 Expression on clinicopathological features of BC Patients

**Fig 4:**
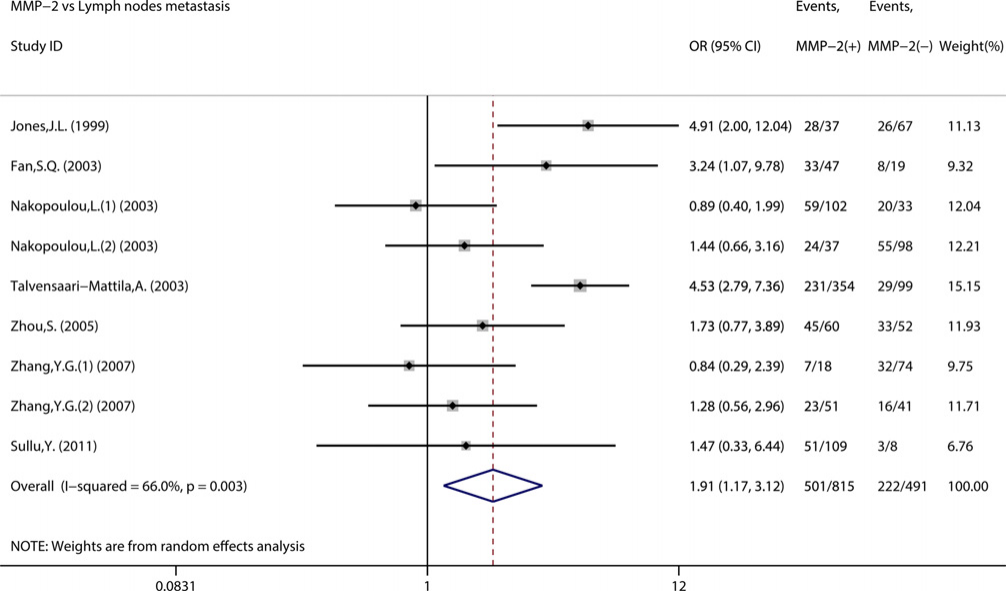
Forest plots of impact of MMP-2 expression on lymph node metastasis.

We assessed the association between MMP-2 expression and clinicopathological features of BC (Table 4). As indicated in Fig. 4, nine studies assessed the association of MMP-2 expression with lymph nodes status. The combined OR was 1.91 (95% CI 1.17–3.12, Z = 2.60, P = 0.009) with heterogeneity (I^2^ = 66.0% P = 0.003). However, no significant association was observed between MMP-2 expression and other clinicopathological features, such as TNM stage, tumor size, histological grade, and so on.

**Table 4:** Meta-analysis of the association between MMP-2 overexpression and clinic-pathological features of breast cancer.

***Stratification of breast cancer***	***No*. *of studies***	***No*. *of patients***	***Analyticalmodel***	***OR(95%CI)***	***Heterogeneity***		
					χ^2^	P	I^2^ (%)
*TNM Stage*	8	938	FEM	1.33 (0.93–1.91)	7.75	0.355	9.70
*Tumor size*	10	1632	FEM	1.20(0.94–1.56)	14.66	0.101	38.60
*Lymph nodes metastasis*	9	1329	REM	1.91(1.17–3.12)	23.54	0.003	66.00
*Distant metastasis*	2	593	FEM	1.42(0.40–5.03)	1.41	0.236	28.90
*Grade*	9	1575	REM	1.47(0.86–2.51)	18.54	0.018	56.90
*ER*	10	1632	REM	0.88(0.62–1.24)	15.08	0.089	40.30
*PR*	9	1492	FEM	0.99(0.77–1.26)	11.44	0.178	30.10

REM, random-effects model; FEM, fixed-effects model; OR, odds ratio; CI, confidence interval.

### 3.4 Publication bias analysis

**Fig 5:**
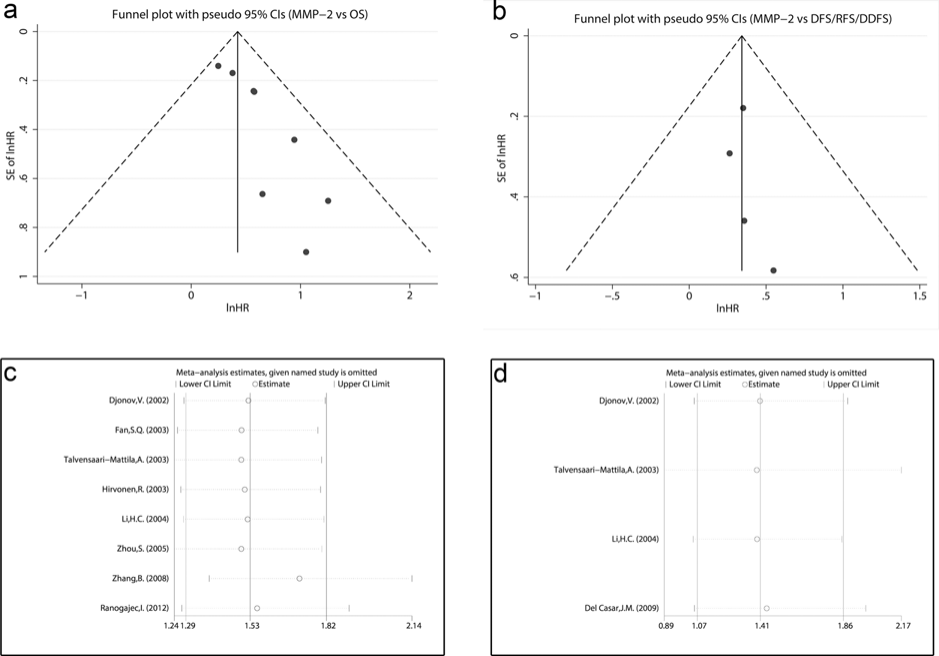
Funnel plots and sensitivity analyses of the meta-analysis. Funnel plots of the meta-analysis assessing (a) MMP-2 expression and OS (b) MMP-2 expression and RFS/DFS. Sensitivity analyses of the meta-analysis assessing (c) MMP-2 expression and OS (d) MMP-2 expression and DFS/RFS/DDFS.

**Fig 6:**
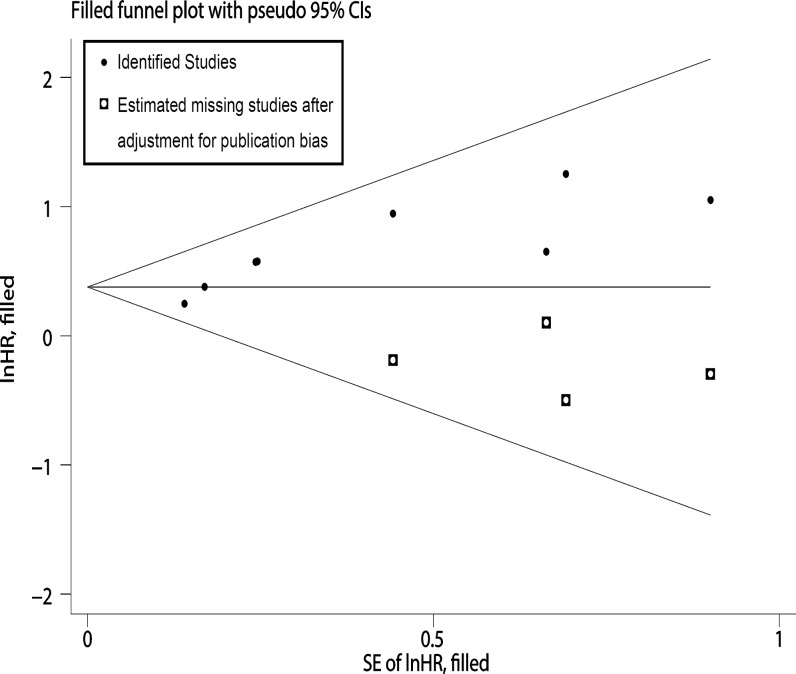
Funnel plots of impact of MMP-2 expression on OS with Trim and Fill method.

Visual inspection of the funnel plot revealed asymmetry in analysis of OS, raising the possibility of publication bias (Fig. 5a). Further estimation using Begg’s test (z = 2.10, P = 0.035) and Egger’s linear regression test (t = 4.3, P = 0.005) also revealed support for significant publication bias. Because of this, we undertook a sensitivity analysis using the trim and fill method, which conservatively imputed hypothetical negative unpublished studies to mirror the positive studies that cause funnel plot asymmetry. The imputed studies produce a symmetrical funnel plot (Fig. 6). The pooled analysis incorporating the hypothetical studies continued to show a statistically significant association between MMP-2 expression and OS (HR = 1.46, 95% CI, 1.27–1.72, z = 4.80, P<0.001).

In analysis of DFS/RFS/DDFS (Fig. 5b), both Begg’s test (z = 0.34, P = 0.734) and Egger’s linear regression test (t = 0.65, P = 0.580) showed no significant publication bias. As for MMP-2 expression and clinicopathological features, no publication bias was observed (S1 Fig.).

### 3.5 Sensitivity analysis

In order to gauge results’ stability, a sensitivity analysis, in which one study was deleted at a time, was performed. No individual study significantly influenced the combined HR of OS or DFS/RFS/DDFS and the combined OR of clinicopathological features by sensitivity analysis, suggesting the robust result of this meta-analysis. The detailed results were shown in Fig. 5c, Fig. 5d and S2 Fig.

## Discussion

BC is the most common cancer in woman with high mortality and its prognosis is still poor despite remarkable advances in treatment [2, 38]. Many studies have shown that MMP-2 is overexpressed in tissues of various human cancer[39], including BC [7]. There are many reports with inconsistent results about the prognostic significance of MMP-2 in BC [14–21, 23–25].

The pooled results from our meta-analysis indicated MMP-2 had a statistical significance for the prognosis of BC, providing strong evidence for applicability of MMP-2 expression in the prognostic analysis of BC.

Subgroup analysis for OS indicated that MMP-2 expression was also significantly associated with poor prognosis in Asian countries or European countries. Significant effects under both multivariate analyses and univariate analyses were also observed. The treatments to the patients among the studies were not exactly the same, which may have an influence on the prognostic value of MMP-2. Subgroup analysis by recruitment time showed consistent tendency although the treatment has been changed during past 20 years all over the world. As for MMP-2 expression and DFS/RFS/DDFS, we didn’t perform a subgroup analysis because of the small sample sizes.

Furthermore, significant correlation was observed between MMP-2 expression and lymph node metastasis, but not other clinocopathological features, such as TNM stage, tumor size, distant metastasis, histological grade, estrogen receptor status and progesterone receptor status. We noted that the results about lymph node metastasis and distant metastasis were inconsistent. Only two studies were available for distant metastasis which may contribute to the discrepant.

In the present study, MMP-2 expression was found in both tumor cells and stromal cells. We also noted that only few studies separately analyzed the significance of stromal accumulation of MMP-2. Usually the stromal expression of MMP-2 was linked with the expression in tumor cells, and their expression was analyzed and graded together as a positive expression. Therefore, the present meta-analysis pooled MMP-2 expression in stromal cells and tumor cells together.

Begg’s tests, Egger’s tests, and funnel plots indicated that publication bias was present in the analysis of association between MMP-2 expression and OS. We performed a quality assessment of the studies to avoid some selection biases and ensure the quality and comparability of studies. We also attempted to minimize publication bias by performing the literature search as complete as possible, using PubMed, Ovid, EMBASE, Web of Science and CNKI. However, our review took into account only fully published studies. Unpublished studies and conference abstracts were not in the scope of our meta-analysis, because the required data were unavailable. In addition to this, our search was restricted to studies published in English or Chinese, due to the fact that other languages were often not accessible to both the authors and readers. Additionally, among the excluded studies, six studies were excluded because of insufficient data. None of the six studies reported significant association between MMP-2 expression and survival in BC. All of the above factors could lead to possible bias and should not be neglected. Nevertheless, the pooled analysis incorporating the hypothetical studies obtained by using the trim and fill method was consistent with the final result, suggesting that the meta-analysis gained approximate the actual results. Moreover, no heterogeneity was observed for the analysis of association between MMP-2 expression and OS.

No publication bias was observed in the analysis of association between MMP-2 expression and DFS/RFS/DDFS and clinicopathological features. Sensitivity analysis showed that omission of any single study did not have significant impact on the combined risk estimates in all of the outcomes. This made the results of this meta-study more reliable to some extent.

Despite our efforts to conduct a comprehensive analysis, some limitations remain to be addressed. Firstly, to minimize the heterogeneity, the included studies were required to measure MMP-2 expression by IHC, which was the most frequently applied method. On one hand, methodological differences of IHC may contribute to heterogeneity. On the other hand, the primary antibody had a significant influence on the sensitivity of IHC. A range of antibodies were used to detect the protein and the dilution of the antibody also differed. Other factors such as storage time and revelation time may also cause potential bias. Subgroup analysis could not be conducted to address these technical problems because few studies offered the concrete data.

Secondly, there was no consistent threshold value to define positive expression in assessment of MMP-2 in BC patients. It is possible that, as a biomarker, the predictive value may also fluctuates along with different amounts/extend of positivity. It cannot be taken into account in this analysis but it is likely that immunoreactivity differs in different staining protocols in different laboratories using perhaps different antibodies. Taken that into account it is almost surprising that the results are this much consistent among the selected studies.

Thirdly, the method of HR estimate required to be stated. For the studies that HR and 95% CI were not reported directly, they were calculated from the available data mentioned in the published articles. If even no available data was provided, we had to extrapolate the value from the survive curve based on the published method [26, 28]. This approach may have caused errors due to the inaccuracies in reading survive curve, so we try our best to minimize errors by reading the curve by two reviewers independently. It seems that the estimated HR may be less trustworthy than that obtained directly. Consequently, we compared the estimated HR and 95% CI with the published results to make sure of the accuracy of the estimated HR.

Lastly, some studies included in the pooled analyses of survival outcomes were analyzed by univariate analysis. Multivariate analysis is more credible than univariate analysis because confounding factors are taken into account. Moreover, different covariates were adjusted in each study, thus leading bias to the pooled results.

Considering these limitations existing in this meta-analysis, our results should be rigorous exposition and the conclusions of this meta-analysis should also be drawn carefully.

In conclusion, our meta-analysis suggests that positive MMP-2 expression is associated with poor survival and lymph nodes metastasis in patients with BC. It is potentially a useful biomarker for predicting prognosis in BC patients. On the basis of our results, larger prospective studies with long-term follow-up are needed by multivariate analysis, which takes into account the well-known prognostic factors in BC.

## Supporting Information

S1 Fig Funnel plots of the meta-analysis assessing MMP-2 expression and clinicopathological features. No publication bias was observed for studies assessing MMP-2 expression and (a) tumor size (b) lymph node metastasis (c) TNM stage (d) histological grade (e) ER status (f) PR status.
(TIF)

S2 Fig Sensitivity analyses of the meta-analysis assessing MMP-2 expression and clinicopathological features. No individual study significantly influenced the combined OR of (a) tumor size (b) lymph node metastasis (c) TNM stage (d) histological grade (e) ER status (f) PR status.
(TIF)

S1 PRISMA checklist (DOC)
