# Supplementary figures and images for: The Impact of Matrix Metalloproteinase 2 on Prognosis and Clinicopathology of Breast Cancer Patients: A Systematic Meta-Analysis
Source: PLoS One. 2015 Mar 27;10(3):e0121404. doi: 10.1371/journal.pone.0121404 (PMC4376789; doi:10.1371/journal.pone.0121404)

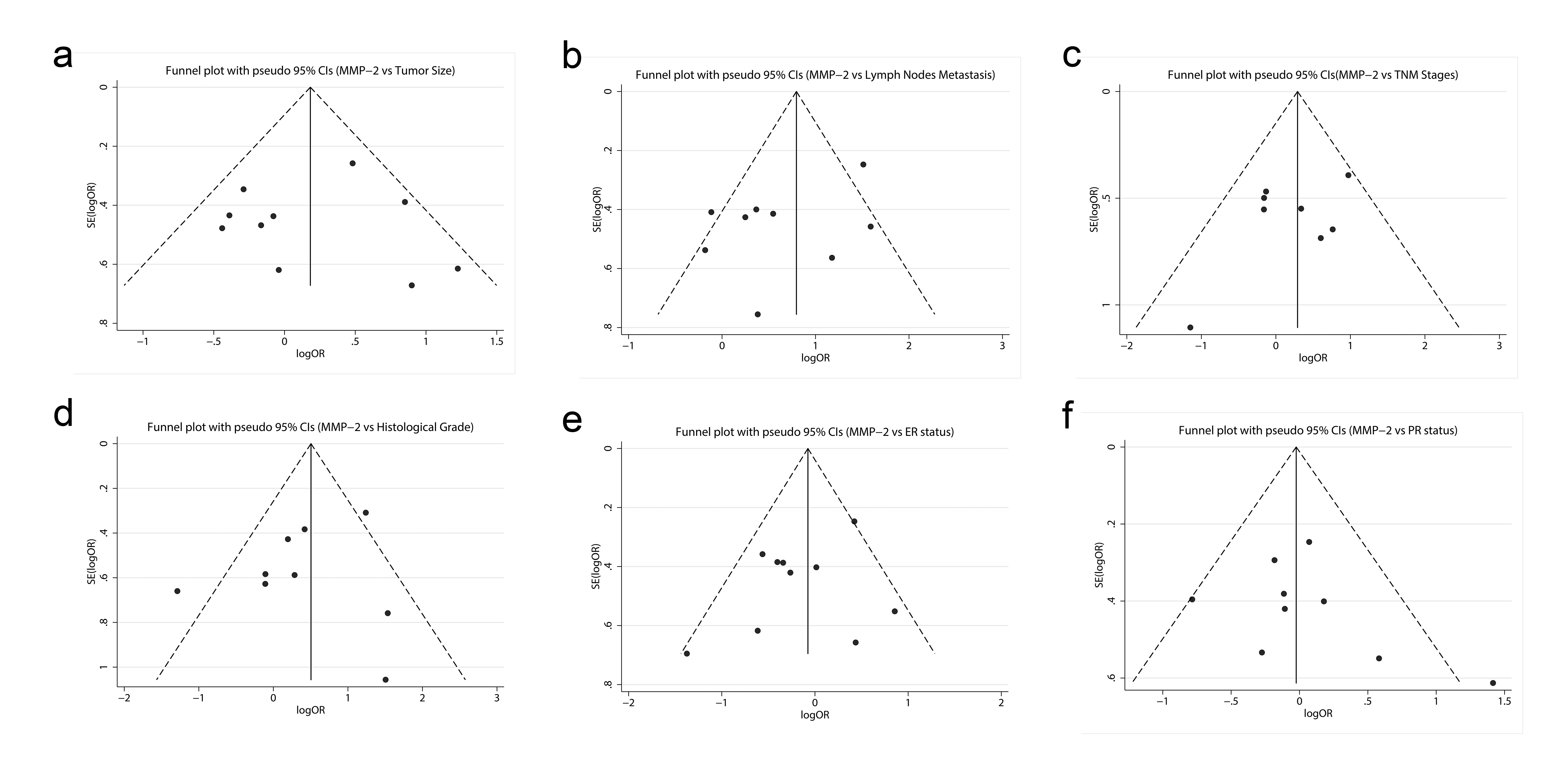

Supplement: S1 Fig — No publication bias was observed for studies assessing MMP-2 expression and (a) tumor size (b) lymph node metastasis (c) TNM stage (d) histological grade (e) ER status (f) PR status. (TIF) [file pone.0121404.s001.tif]

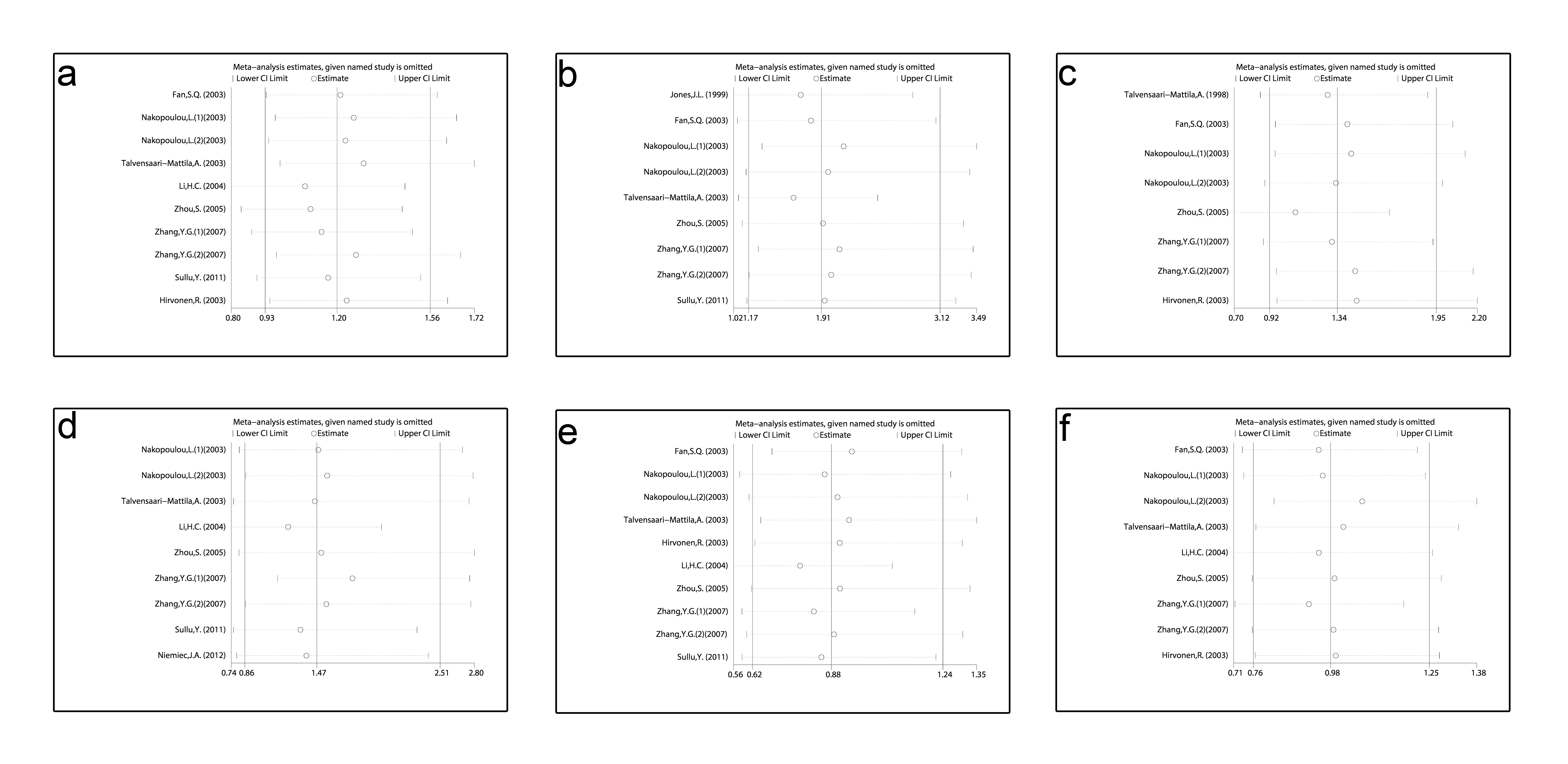

Supplement: S2 Fig — No individual study significantly influenced the combined OR of (a) tumor size (b) lymph node metastasis (c) TNM stage (d) histological grade (e) ER status (f) PR status. (TIF) [file pone.0121404.s002.tif]
